# Crystal structure and DFT study of benzyl 1-benzyl-2-oxo-1,2-di­hydro­quinoline-4-carboxyl­ate

**DOI:** 10.1107/S2056989019007989

**Published:** 2019-06-11

**Authors:** Younos Bouzian, Md. Serajul Haque Faizi, Joel T. Mague, Bouchaib El Otmani, Necmi Dege, Khalid Karrouchi, El Mokhtar Essassi

**Affiliations:** a Laboratoire de Chimie Organique Hétérocyclique, Centre de Recherche Des Sciences des Médicaments, Pôle de Compétence Pharmacochimie, Av Ibn Battouta, BP 1014, Faculté des Sciences, Université Mohammed V, Rabat, Morocco; b Department of Chemistry, Langat Singh College, Babasaheb Bhimrao Ambedkar Bihar University, Muzaffarpur, Bihar-842001, India; c Department of Chemistry, Tulane University, New Orleans, LA 70118, USA; d Ondokuz Mayıs University, Faculty of Arts and Sciences, Department of Physics, 55139, Kurupelit, Samsun, Turkey; e Laboratory of Plant Chemistry, Organic and Bioorganic Synthesis, URAC23, Faculty of Science, BP 1014, GEOPAC Research Center, Mohammed V University, Rabat, Morocco

**Keywords:** crystal structure, 2-oxo-1,2-di­hydro­quinoline, C—H⋯O hydrogen bonding, C—H⋯π inter­actions, supra­molecular three-dimensional structure, DFT

## Abstract

In the title quinoline derivative, C_24_H_19_NO_3_, the two benzyl rings are inclined to the quinoline ring mean plane by 74.09 (8) and 89.43 (7)°, and to each other by 63.97 (10)°. The carboxyl­ate group is twisted from the quinoline ring mean plane by 32.2 (2)°. There is a short intra­molecular C—H⋯O contact forming an *S*(6) ring motif. In the crystal, mol­ecules are linked by bifurcated C—H,H⋯O hydrogen bonds, forming layers parallel to the *ac* plane. The layers are linked by C—H⋯π inter­actions, forming a supra­molecular three-dimensional structure.

## Abstract 2

In the title quinoline derivative, the two benzyl rings are inclined to the quinoline ring mean plane by 74.09 (8) and 89.43 (7)°.

## Chemical context   

Heterocyclic compounds have paved the way for exceptional achievements in the fight against many life-threatening diseases (Alcaide *et al.*, 2010 ▸). It is therefore no surprise that the development of new methodologies to synthesize biologically active heterocyclic compounds persists as a very important goal in organic chemistry (Jones *et al.*, 2011 ▸). Quinolones and their derivatives have contributed substanti­ally to the evolution of anti­microbial agents. The development of anti­biotic quinolone began in 1962 with the discovery of nalidixic acid, which was used to treat urinary tract infections (Lesher *et al.*, 1962 ▸). Quinolone derivatives are a classical division of organic chemistry; many of these mol­ecules have shown remarkable biological properties, including exceptional anti­bacterial activity (Beena & Rawat, 2013 ▸; Chai *et al.*, 2011 ▸; Hoshino *et al.*, 2008 ▸) and are used as anti-fungal (Musiol *et al.*, 2010 ▸), anti-tumoral (Bergh *et al.*, 1997 ▸) and anti-cancer drugs (Elderfield & LeVon, 1960 ▸). Recently, complexes based on quinoline-4-carb­oxy­lic acid have been reported (Bu *et al.*, 2005 ▸; Xiong *et al.*, 2000 ▸). The present study is a continuation of the synthesis of heterocyclic derivatives performed by our team (Chkirate *et al.*, 2019*a* ▸,*b* ▸). It is part of an ongoing structural study of heterocyclic compounds and their utilization as mol­ecular (Faizi *et al.*, 2016 ▸) and fluorescence sensors (Mukherjee *et al.*, 2018 ▸); Kumar *et al.*, 2017 ▸, 2018 ▸). We report herein the synthesis and the mol­ecular and crystal structures of the title compound, benzyl 1-benzyl-2-oxo-1,2-di­hydro­quinoline-4-carboxyl­ate, along with the density functional theory (DFT) calculations.
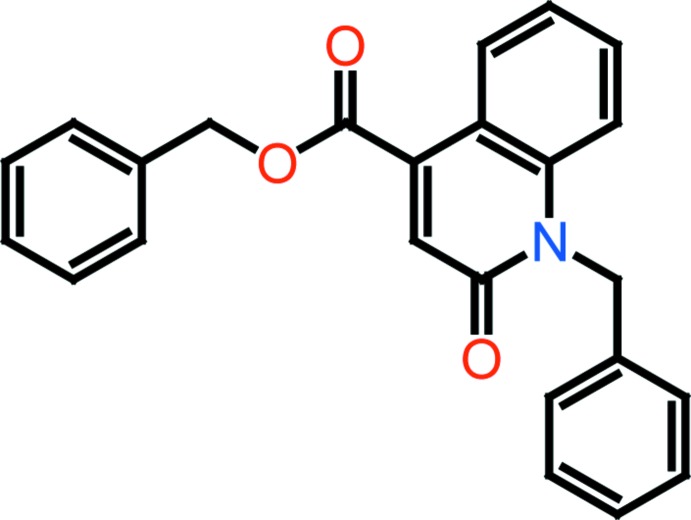


## Structural commentary   

**Figure 1:**
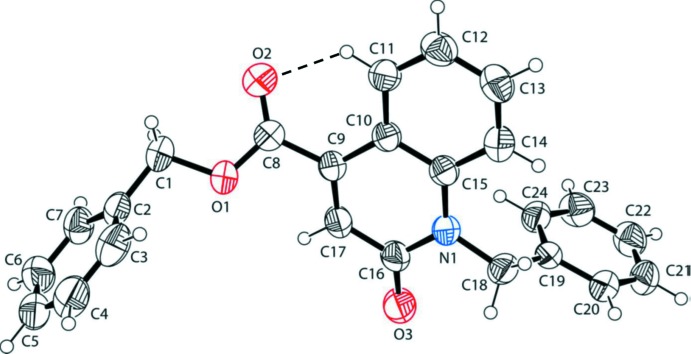
A view of the mol­ecular structure of the title compound, with the atom labelling. Displacement ellipsoids are drawn at the 40% probability level. The intra­molecular C—H⋯O contact (see Table 1 ▸) is shown as a dashed line.

The mol­ecular structure of the title compound is illustrated in Fig. 1 ▸. It is composed of two substituted aromatic rings attached to a planar quinolone ring (N1/C9–C17; r.m.s. deviation = 0.017 Å). The attached benzyl rings (C2–C7 and C19–C24) are inclined to the quinolone ring system by 74.09 (8) and 89.43 (7)°, respectively, and to each other by 63.97 (10)°. The carboxyl­ate group is twisted from the quinoline ring system by 32.2 (2)°. The carboxyl­ate group is involved in a short intra­molecular C—H⋯O contact forming an *S*(6) ring motif (Fig. 1 ▸ and Table 1 ▸).

## Supra­molecular features   

**Figure 2:**
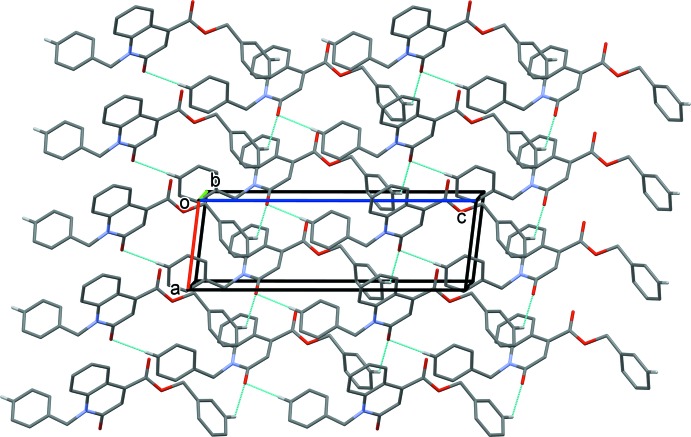
A view of along the *b* axis of the crystal packing of the title compound. Hydrogen bonds (see Table 1 ▸) are shown as dashed lines. For clarity, only H atoms H6 and H22 have been included.

**Figure 3:**
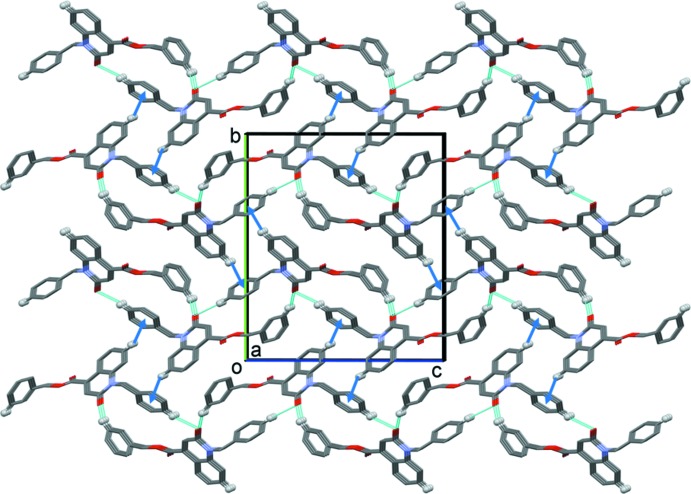
A view of along the *c* axis of the crystal packing of the title compound. Hydrogen bonds are shown as dashed lines and the C—H⋯π inter­actions as blue arrows (see Table 1 ▸). For clarity, only H atoms H6, H22 and H13 have been included (as grey balls).

In the crystal, mol­ecules are linked by bifurcated C—H,H⋯O hydrogen bonds, forming layers lying parallel to the *ac* plane (Table 1 ▸ and Fig. 2 ▸). The layers are linked by C—H··*·π* inter­actions, so forming a supra­molecular three-dimensional structure (Table 1 ▸ and Fig. 3 ▸).

## Frontier mol­ecular orbital analysis   

**Figure 4:**
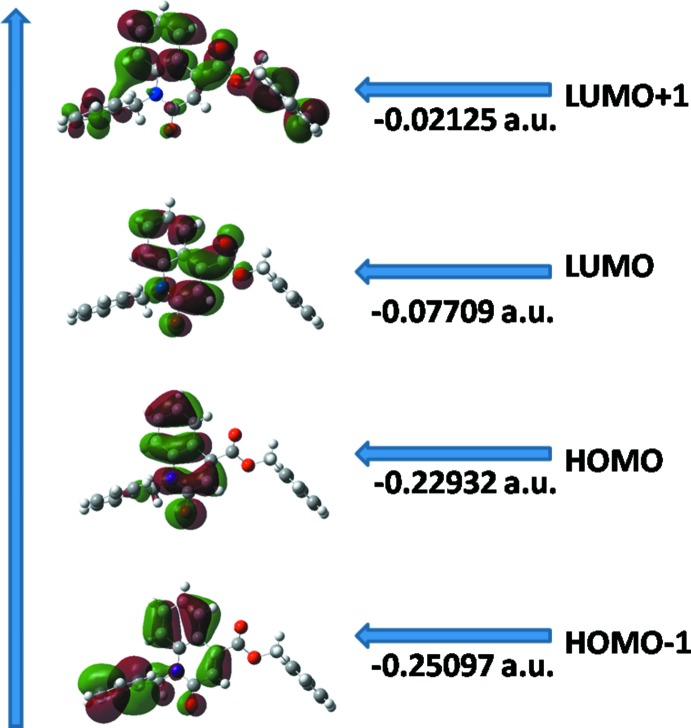
Electron distribution of the HOMO-1, HOMO, LUMO and the LUMO+1 energy levels for the title compound.

The highest occupied mol­ecular orbitals (HOMOs) and the lowest unoccupied mol­ecular orbitals (LUMOs) are named as frontier mol­ecular orbitals (FMOs). The FMOs play an important role in the optical and electric properties, as well as in quantum chemistry and UV–Vis spectra. The frontier orbital gap helps characterize the chemical reactivity and the kinetic stability of the mol­ecule. A mol­ecule with a small frontier orbital gap is generally associated with a high chemical reactivity, low kinetic stability and is also termed a soft mol­ecule. DFT quantum-chemical calculations for the title compound were performed at the B3LYP/6–311 G(d,p) level (Becke, 1993 ▸) as implemented in *GAUSSIAN09* (Frisch *et al.*, 2009 ▸). DFT structure optimization was performed starting from the X-ray geometry and the values compared with experimental values of bond lengths and bond angles matching with theoretical values. The basis set 6-311G(d,p) is well suited in its approach to the experimental data. The DFT study shows that the HOMO and LUMO are localized in the plane extending from the whole tetra-substituted benzene ring. The electron distribution of the HOMO-1, HOMO, LUMO and the LUMO+1 energy levels are shown in Fig. 4 ▸. The HOMO mol­ecular orbital exhibits both *σ* and *π* character, whereas HOMO-1 is dominated by *π*-orbital density. The LUMO is mainly composed of *π*-density while LUMO+1 has both *σ* and *π* electronic density. The HOMO–LUMO gap is found to be 0.15223 a.u. and the frontier mol­ecular orbital energies, *E*_HOMO_ and *E*_LUMO_ are −0.22932 and −0.07709 a.u., respectively.

## Database survey   

**Figure 5:**
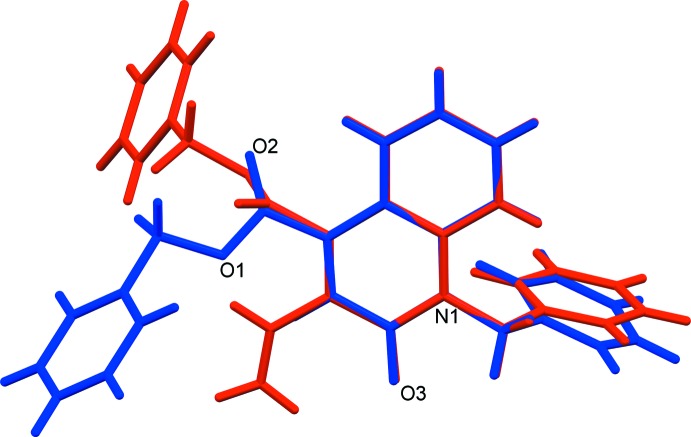
The structural overlap (*Mercury*; Macrae *et al.*, 2008 ▸) of the title compound (blue) and FAVZEK (red: benzyl 1-benzyl-2-oxo-3-vinyl-1,2-di­hydro­quinoline-4-carboxyl­ate; Malini *et al.*, 2017 ▸).

A search of the Cambridge Structural Database (CSD, version 5.40, update May 2019; Groom *et al.*, 2016 ▸) for the 1-benzyl­quinolin-2(1*H*)-one skeleton gave ten hits. The dihedral angle between the benzyl and quinoline rings varies from *ca* 71.0 to 89.6°, compared to 89.43 (7)° in the title compound. Only two of these compounds have a carboxyl­ate group in position 4 on the quinoline ring, *viz*. ethyl 1-benzyl-3-hy­droxy-2-oxo-1,2-di­hydro­quinoline-4-carboxyl­ate (CSD refcode ZINHEL; Paterna *et al.*, 2013 ▸) and benzyl 1-benzyl-2-oxo-3-vinyl-1,2-di­hydro­quinoline-4-carboxyl­ate (FAVZEK; Malini *et al.*, 2017 ▸). The latter compound most closely resembles the title compound, with a vinyl substituent in position 3 of the quinoline ring. A view of the structural overlap of FAVZEK and the title compound is given in Fig. 5 ▸. The conformation of the two compounds differs essentially in the orientation of the carboxyl­ate group with respect to the quinoline ring: 85.6 (3)° in FAVZEK compared to 32.2 (2)° in the title compound. This is the result of steric hindrance resulting from the presence of the vinyl substituent in position 3 on the quinoline ring in FAVZEK. In the title compound, the benzyl rings (C19–C24 and C2–C7) are inclined to the quinoline ring by 89.43 (7) and 74.09 (8)°, respectively, while in FAVZEK the corresponding dihedral angles are 88.55 (11) and 76.44 (13)°. The two benzyl rings are inclined to each other by 63.97 (10)° in the title compound compared to 73.38 (16)° in FAVZEK.

## Synthesis and crystallization   

A mixture of 2-oxo-1,2-di­hydro­quinoline-4-carb­oxy­lic acid (1 g, 5.29 mmol), K_2_CO_3_ (1.46 g, 10.58 mmol), benzyl chloride (1.21 ml, 10.58 mmol) and tetra *n*-butyl­ammonium bromide as catalyst in DMF (50 ml) was stirred at room temperature for 48 h. The solution was filtered by suction and the solvent was removed under reduced pressure. The residue was chromatographed on a silica-gel column using hexane and ethyl acetate (*v*/*v*, 95/5) as eluents to afford the title compound. Colourless prismatic crystals of the title compound were obtained by slow evaporation of a solution in ethanol (yield 53%).

## Refinement   

Crystal data, data collection and structure refinement details are summarized in Table 2 ▸. The C-bound H atoms were placed in calculated positions and included in the refinement in the riding-model approximation: C—H = 0.93–0.97 Å with *U*_iso_(H) = 1.2*U*_eq_(C).

## Supplementary Material

Crystal structure: contains datablock(s) I, Global. DOI: 10.1107/S2056989019007989/su5500sup1.cif

Structure factors: contains datablock(s) I. DOI: 10.1107/S2056989019007989/su5500Isup2.hkl

Supporting information file. DOI: 10.1107/S2056989019007989/su5500Isup3.cml

CCDC reference: 1920509

Additional supporting information:  crystallographic information; 3D view; checkCIF report

## Figures and Tables

**Table 1:** Hydrogen-bond geometry (Å, °) *Cg*1 is the centroid of the C19–C24 ring.

*D*—H⋯*A*	*D*—H	H⋯*A*	*D*⋯*A*	*D*—H⋯*A*
C11—H11⋯O2	0.93	2.34	2.962 (2)	124
C6—H6⋯O3^i^	0.93	2.55	3.184 (3)	126
C22—H22⋯O3^ii^	0.93	2.56	3.490 (2)	174
C13—H13⋯*Cg*1^iii^	0.93	2.91	3.727 (2)	147

Symmetry codes: (i) 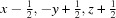; (ii) 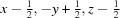; (iii) 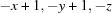.

**Table 2:** Experimental details

Crystal data	
Chemical formula	C_24_H_19_NO_3_
*M*_r_	369.40
Crystal system, space group	Monoclinic, *P*2_1_/*n*
Temperature (K)	296
*a*, *b*, *c* (Å)	5.6101 (4), 19.5523 (11), 17.2761 (11)
β (°)	96.969 (5)
*V* (Å^3^)	1881.0 (2)
*Z*	4
Radiation type	Mo *K*α
μ (mm^−1^)	0.09
Crystal size (mm)	0.71 × 0.52 × 0.25

Data collection	
Diffractometer	STOE IPDS 2
Absorption correction	Integration (*X-RED32*; Stoe & Cie, 2002 ▸)
*T*_min_, *T*_max_	0.949, 0.979
No. of measured, independent and observed [*I* > 2σ(*I*)] reflections	15909, 3686, 2270
*R*_int_	0.046
(sin θ/λ)_max_ (Å^−1^)	0.617

Refinement	
*R*[*F* ^2^ > 2σ(*F* ^2^)], *wR*(*F* ^2^), *S*	0.041, 0.115, 0.95
No. of reflections	3686
No. of parameters	254
H-atom treatment	H-atom parameters constrained
Δρ_max_, Δρ_min_ (e Å^−3^)	0.29, −0.17

Computer programs: *X-AREA* and *X-RED32* (Stoe & Cie, 2002 ▸), *SHELXT2018/2* (Sheldrick, 2015*a* ▸), *SHELXL2018/3* (Sheldrick, 2015*b* ▸), *ORTEP-3 for Windows* and *WinGX* (Farrugia, 2012 ▸), *Mercury* (Macrae *et al.*, 2008 ▸) and *PLATON* (Spek, 2009 ▸).

## supplementary crystallographic information

### Crystal data

**Table:**

C_24_H_19_NO_3_	*F*(000) = 776
*M**_r_* = 369.40	*D*_x_ = 1.304 Mg m^−^^3^
Monoclinic, *P*2_1_/*n*	Mo *K*α radiation, λ = 0.71073 Å
*a* = 5.6101 (4) Å	Cell parameters from 14721 reflections
*b* = 19.5523 (11) Å	θ = 2.1–30.9°
*c* = 17.2761 (11) Å	µ = 0.09 mm^−^^1^
β = 96.969 (5)°	*T* = 296 K
*V* = 1881.0 (2) Å^3^	Prism, colourless
*Z* = 4	0.71 × 0.52 × 0.25 mm

### Data collection

**Table:**

STOE IPDS 2 diffractometer	3686 independent reflections
Radiation source: sealed X-ray tube, 12 x 0.4 mm long-fine focus	2270 reflections with *I* > 2σ(*I*)
Plane graphite monochromator	*R*_int_ = 0.046
Detector resolution: 6.67 pixels mm^-1^	θ_max_ = 26.0°, θ_min_ = 2.1°
rotation method scans	*h* = −6→6
Absorption correction: integration (X-RED32; Stoe & Cie, 2002)	*k* = −24→24
*T*_min_ = 0.949, *T*_max_ = 0.979	*l* = −21→21
15909 measured reflections	

### Refinement

**Table:**

Refinement on *F*^2^	Secondary atom site location: difference Fourier map
Least-squares matrix: full	Hydrogen site location: inferred from neighbouring sites
*R*[*F*^2^ > 2σ(*F*^2^)] = 0.041	H-atom parameters constrained
*wR*(*F*^2^) = 0.115	*w* = 1/[σ^2^(*F*_o_^2^) + (0.0653*P*)^2^] where *P* = (*F*_o_^2^ + 2*F*_c_^2^)/3
*S* = 0.95	(Δ/σ)_max_ < 0.001
3686 reflections	Δρ_max_ = 0.29 e Å^−^^3^
254 parameters	Δρ_min_ = −0.17 e Å^−^^3^
0 restraints	Extinction correction: (SHELXL2018/3; Sheldrick, 2015b), Fc^*^=kFc[1+0.001xFc^2^λ^3^/sin(2θ)]^-1/4^
Primary atom site location: structure-invariant direct methods	Extinction coefficient: 0.0160 (19)

### Special details

**Table:**

Geometry. All esds (except the esd in the dihedral angle between two l.s. planes) are estimated using the full covariance matrix. The cell esds are taken into account individually in the estimation of esds in distances, angles and torsion angles; correlations between esds in cell parameters are only used when they are defined by crystal symmetry. An approximate (isotropic) treatment of cell esds is used for estimating esds involving l.s. planes.

### Fractional atomic coordinates and isotropic or equivalent isotropic displacement parameters (Å^2^)

**Table:**

	*x*	*y*	*z*	*U*_iso_*/*U*_eq_	
O1	0.6381 (2)	0.38420 (8)	0.44614 (6)	0.0805 (4)	
O2	0.2849 (3)	0.41941 (9)	0.38836 (8)	0.0926 (5)	
O3	1.0727 (3)	0.30369 (9)	0.23941 (8)	0.0974 (5)	
N1	0.8617 (2)	0.39351 (7)	0.18321 (7)	0.0571 (4)	
C1	0.5448 (4)	0.38554 (13)	0.52105 (10)	0.0808 (6)	
H1A	0.423278	0.350492	0.522931	0.097*	
H1B	0.473201	0.429694	0.529330	0.097*	
C2	0.7519 (3)	0.37265 (9)	0.58196 (9)	0.0603 (4)	
C3	0.9437 (4)	0.41769 (11)	0.59339 (12)	0.0802 (6)	
H3	0.945035	0.456570	0.562433	0.096*	
C4	1.1323 (4)	0.40511 (13)	0.65041 (13)	0.0906 (7)	
H4	1.260989	0.435378	0.657408	0.109*	
C5	1.1318 (4)	0.34884 (15)	0.69647 (12)	0.0909 (7)	
H5	1.260436	0.340420	0.734504	0.109*	
C6	0.9439 (4)	0.30506 (12)	0.68696 (12)	0.0835 (6)	
H6	0.942364	0.266986	0.719161	0.100*	
C7	0.7553 (3)	0.31656 (10)	0.63000 (10)	0.0693 (5)	
H7	0.627693	0.285877	0.623838	0.083*	
C8	0.4909 (3)	0.40350 (9)	0.38491 (10)	0.0627 (5)	
C9	0.6101 (3)	0.40005 (8)	0.31252 (9)	0.0542 (4)	
C10	0.5488 (3)	0.44685 (8)	0.24814 (9)	0.0512 (4)	
C11	0.3727 (3)	0.49795 (9)	0.24800 (10)	0.0608 (4)	
H11	0.284034	0.501326	0.289959	0.073*	
C12	0.3296 (3)	0.54270 (9)	0.18732 (11)	0.0704 (5)	
H12	0.214571	0.576813	0.188525	0.085*	
C13	0.4568 (4)	0.53723 (10)	0.12432 (11)	0.0729 (5)	
H13	0.426062	0.567624	0.082904	0.088*	
C14	0.6281 (3)	0.48765 (9)	0.12175 (10)	0.0652 (5)	
H14	0.710104	0.484076	0.078274	0.078*	
C15	0.6801 (3)	0.44252 (8)	0.18394 (9)	0.0526 (4)	
C16	0.9158 (3)	0.34674 (10)	0.24281 (10)	0.0669 (5)	
C17	0.7823 (3)	0.35337 (10)	0.30827 (9)	0.0660 (5)	
H17	0.817545	0.323540	0.350029	0.079*	
C18	1.0159 (3)	0.39223 (10)	0.12080 (9)	0.0632 (5)	
H18A	1.169995	0.372994	0.141437	0.076*	
H18B	1.043763	0.438967	0.105353	0.076*	
C19	0.9192 (3)	0.35221 (8)	0.04907 (9)	0.0516 (4)	
C20	1.0478 (3)	0.35411 (10)	−0.01382 (10)	0.0647 (5)	
H20	1.185150	0.381033	−0.011516	0.078*	
C21	0.9761 (4)	0.31666 (11)	−0.08022 (10)	0.0767 (6)	
H21	1.066000	0.318117	−0.122010	0.092*	
C22	0.7736 (4)	0.27749 (11)	−0.08482 (11)	0.0791 (6)	
H22	0.724133	0.252641	−0.129819	0.095*	
C23	0.6436 (4)	0.27501 (10)	−0.02272 (12)	0.0784 (6)	
H23	0.505989	0.248180	−0.025486	0.094*	
C24	0.7161 (3)	0.31221 (10)	0.04413 (10)	0.0644 (5)	
H24	0.626799	0.310163	0.086046	0.077*	

### Atomic displacement parameters (Å^2^)

**Table:**

	*U*^11^	*U*^22^	*U*^33^	*U*^12^	*U*^13^	*U*^23^
O1	0.0716 (8)	0.1257 (12)	0.0459 (6)	0.0180 (8)	0.0138 (6)	0.0066 (7)
O2	0.0757 (9)	0.1299 (13)	0.0751 (9)	0.0236 (9)	0.0211 (7)	0.0157 (9)
O3	0.1090 (11)	0.1200 (12)	0.0646 (8)	0.0597 (10)	0.0158 (7)	0.0014 (8)
N1	0.0554 (8)	0.0715 (9)	0.0438 (7)	0.0039 (7)	0.0038 (6)	−0.0072 (6)
C1	0.0775 (12)	0.1182 (17)	0.0503 (10)	0.0091 (12)	0.0225 (9)	0.0059 (11)
C2	0.0656 (10)	0.0720 (11)	0.0462 (8)	−0.0049 (9)	0.0191 (8)	−0.0062 (8)
C3	0.0999 (15)	0.0716 (12)	0.0756 (13)	−0.0163 (11)	0.0368 (12)	−0.0042 (10)
C4	0.0796 (13)	0.1186 (19)	0.0761 (14)	−0.0394 (13)	0.0196 (12)	−0.0306 (14)
C5	0.0716 (12)	0.147 (2)	0.0550 (11)	−0.0068 (14)	0.0100 (10)	−0.0096 (13)
C6	0.0852 (14)	0.1003 (16)	0.0652 (12)	−0.0016 (12)	0.0099 (10)	0.0125 (11)
C7	0.0771 (12)	0.0755 (12)	0.0564 (10)	−0.0184 (10)	0.0129 (9)	−0.0005 (9)
C8	0.0591 (10)	0.0727 (12)	0.0572 (10)	0.0062 (9)	0.0102 (8)	−0.0003 (8)
C9	0.0546 (9)	0.0624 (10)	0.0448 (8)	0.0017 (8)	0.0031 (7)	−0.0039 (7)
C10	0.0505 (8)	0.0558 (9)	0.0460 (8)	−0.0026 (7)	0.0005 (7)	−0.0083 (7)
C11	0.0606 (10)	0.0634 (10)	0.0574 (9)	0.0047 (8)	0.0031 (8)	−0.0092 (8)
C12	0.0773 (12)	0.0599 (11)	0.0718 (12)	0.0133 (9)	−0.0002 (10)	−0.0007 (9)
C13	0.0873 (13)	0.0644 (12)	0.0652 (11)	0.0039 (10)	0.0016 (10)	0.0111 (9)
C14	0.0729 (11)	0.0687 (11)	0.0545 (9)	−0.0053 (9)	0.0099 (8)	0.0024 (8)
C15	0.0518 (8)	0.0562 (10)	0.0485 (9)	−0.0034 (7)	0.0008 (7)	−0.0062 (7)
C16	0.0697 (11)	0.0811 (12)	0.0483 (9)	0.0213 (10)	0.0014 (8)	−0.0065 (9)
C17	0.0751 (11)	0.0771 (12)	0.0448 (9)	0.0178 (10)	0.0039 (8)	0.0025 (8)
C18	0.0529 (9)	0.0834 (13)	0.0539 (9)	−0.0039 (8)	0.0092 (8)	−0.0107 (9)
C19	0.0493 (8)	0.0602 (10)	0.0450 (8)	0.0077 (7)	0.0049 (7)	0.0009 (7)
C20	0.0618 (10)	0.0778 (12)	0.0558 (10)	0.0031 (9)	0.0125 (8)	0.0002 (9)
C21	0.0878 (13)	0.0937 (15)	0.0506 (10)	0.0169 (12)	0.0167 (9)	−0.0051 (10)
C22	0.0912 (14)	0.0853 (14)	0.0585 (11)	0.0099 (12)	−0.0011 (11)	−0.0193 (10)
C23	0.0741 (12)	0.0816 (14)	0.0764 (13)	−0.0097 (10)	−0.0036 (11)	−0.0154 (11)
C24	0.0603 (10)	0.0794 (12)	0.0543 (9)	−0.0037 (9)	0.0101 (8)	−0.0041 (9)

### Geometric parameters (Å, º)

**Table:**

O1—C8	1.316 (2)	C10—C15	1.406 (2)
O1—C1	1.454 (2)	C11—C12	1.364 (2)
O2—C8	1.205 (2)	C11—H11	0.9300
O3—C16	1.225 (2)	C12—C13	1.376 (3)
N1—C16	1.383 (2)	C12—H12	0.9300
N1—C15	1.400 (2)	C13—C14	1.369 (3)
N1—C18	1.462 (2)	C13—H13	0.9300
C1—C2	1.491 (3)	C14—C15	1.394 (2)
C1—H1A	0.9700	C14—H14	0.9300
C1—H1B	0.9700	C16—C17	1.436 (3)
C2—C7	1.374 (2)	C17—H17	0.9300
C2—C3	1.386 (3)	C18—C19	1.510 (2)
C3—C4	1.378 (3)	C18—H18A	0.9700
C3—H3	0.9300	C18—H18B	0.9700
C4—C5	1.358 (3)	C19—C24	1.376 (2)
C4—H4	0.9300	C19—C20	1.376 (2)
C5—C6	1.352 (3)	C20—C21	1.379 (3)
C5—H5	0.9300	C20—H20	0.9300
C6—C7	1.373 (3)	C21—C22	1.364 (3)
C6—H6	0.9300	C21—H21	0.9300
C7—H7	0.9300	C22—C23	1.369 (3)
C8—C9	1.490 (2)	C22—H22	0.9300
C9—C17	1.338 (2)	C23—C24	1.383 (2)
C9—C10	1.449 (2)	C23—H23	0.9300
C10—C11	1.405 (2)	C24—H24	0.9300
			
C8—O1—C1	116.85 (14)	C11—C12—H12	120.1
C16—N1—C15	122.69 (14)	C13—C12—H12	120.1
C16—N1—C18	116.21 (14)	C14—C13—C12	120.92 (17)
C15—N1—C18	120.98 (14)	C14—C13—H13	119.5
O1—C1—C2	106.92 (15)	C12—C13—H13	119.5
O1—C1—H1A	110.3	C13—C14—C15	120.37 (17)
C2—C1—H1A	110.3	C13—C14—H14	119.8
O1—C1—H1B	110.3	C15—C14—H14	119.8
C2—C1—H1B	110.3	C14—C15—N1	120.74 (16)
H1A—C1—H1B	108.6	C14—C15—C10	119.28 (15)
C7—C2—C3	117.88 (17)	N1—C15—C10	119.98 (14)
C7—C2—C1	120.90 (17)	O3—C16—N1	120.85 (17)
C3—C2—C1	121.20 (18)	O3—C16—C17	123.13 (17)
C4—C3—C2	120.3 (2)	N1—C16—C17	116.00 (15)
C4—C3—H3	119.9	C9—C17—C16	123.63 (16)
C2—C3—H3	119.9	C9—C17—H17	118.2
C5—C4—C3	120.5 (2)	C16—C17—H17	118.2
C5—C4—H4	119.8	N1—C18—C19	115.36 (13)
C3—C4—H4	119.8	N1—C18—H18A	108.4
C6—C5—C4	119.9 (2)	C19—C18—H18A	108.4
C6—C5—H5	120.0	N1—C18—H18B	108.4
C4—C5—H5	120.0	C19—C18—H18B	108.4
C5—C6—C7	120.3 (2)	H18A—C18—H18B	107.5
C5—C6—H6	119.8	C24—C19—C20	118.42 (16)
C7—C6—H6	119.8	C24—C19—C18	123.92 (15)
C6—C7—C2	121.09 (18)	C20—C19—C18	117.62 (15)
C6—C7—H7	119.5	C19—C20—C21	120.96 (18)
C2—C7—H7	119.5	C19—C20—H20	119.5
O2—C8—O1	123.11 (17)	C21—C20—H20	119.5
O2—C8—C9	125.75 (16)	C22—C21—C20	120.22 (19)
O1—C8—C9	111.09 (14)	C22—C21—H21	119.9
C17—C9—C10	119.64 (15)	C20—C21—H21	119.9
C17—C9—C8	118.55 (15)	C21—C22—C23	119.53 (18)
C10—C9—C8	121.79 (14)	C21—C22—H22	120.2
C11—C10—C15	118.48 (15)	C23—C22—H22	120.2
C11—C10—C9	123.49 (15)	C22—C23—C24	120.35 (19)
C15—C10—C9	117.97 (14)	C22—C23—H23	119.8
C12—C11—C10	121.10 (17)	C24—C23—H23	119.8
C12—C11—H11	119.5	C19—C24—C23	120.51 (18)
C10—C11—H11	119.5	C19—C24—H24	119.7
C11—C12—C13	119.81 (17)	C23—C24—H24	119.7
			
C8—O1—C1—C2	−171.20 (17)	C16—N1—C15—C14	−178.53 (15)
O1—C1—C2—C7	−118.66 (19)	C18—N1—C15—C14	5.5 (2)
O1—C1—C2—C3	63.1 (2)	C16—N1—C15—C10	1.9 (2)
C7—C2—C3—C4	1.2 (3)	C18—N1—C15—C10	−174.07 (14)
C1—C2—C3—C4	179.46 (18)	C11—C10—C15—C14	−1.5 (2)
C2—C3—C4—C5	−0.6 (3)	C9—C10—C15—C14	−178.87 (14)
C3—C4—C5—C6	−0.6 (3)	C11—C10—C15—N1	178.12 (13)
C4—C5—C6—C7	1.2 (3)	C9—C10—C15—N1	0.7 (2)
C5—C6—C7—C2	−0.5 (3)	C15—N1—C16—O3	178.49 (17)
C3—C2—C7—C6	−0.7 (3)	C18—N1—C16—O3	−5.4 (2)
C1—C2—C7—C6	−178.97 (19)	C15—N1—C16—C17	−3.1 (2)
C1—O1—C8—O2	−2.7 (3)	C18—N1—C16—C17	173.04 (15)
C1—O1—C8—C9	179.62 (17)	C10—C9—C17—C16	0.7 (3)
O2—C8—C9—C17	−147.7 (2)	C8—C9—C17—C16	−177.71 (16)
O1—C8—C9—C17	29.9 (2)	O3—C16—C17—C9	−179.80 (19)
O2—C8—C9—C10	33.9 (3)	N1—C16—C17—C9	1.8 (3)
O1—C8—C9—C10	−148.47 (15)	C16—N1—C18—C19	97.36 (17)
C17—C9—C10—C11	−179.20 (16)	C15—N1—C18—C19	−86.45 (19)
C8—C9—C10—C11	−0.9 (2)	N1—C18—C19—C24	−7.8 (2)
C17—C9—C10—C15	−2.0 (2)	N1—C18—C19—C20	174.72 (15)
C8—C9—C10—C15	176.37 (14)	C24—C19—C20—C21	−0.3 (3)
C15—C10—C11—C12	−0.2 (2)	C18—C19—C20—C21	177.36 (16)
C9—C10—C11—C12	176.97 (15)	C19—C20—C21—C22	0.7 (3)
C10—C11—C12—C13	1.2 (3)	C20—C21—C22—C23	−0.7 (3)
C11—C12—C13—C14	−0.5 (3)	C21—C22—C23—C24	0.3 (3)
C12—C13—C14—C15	−1.3 (3)	C20—C19—C24—C23	−0.1 (3)
C13—C14—C15—N1	−177.34 (16)	C18—C19—C24—C23	−177.59 (17)
C13—C14—C15—C10	2.3 (2)	C22—C23—C24—C19	0.1 (3)

### Hydrogen-bond geometry (Å, º)

Cg1 is the centroid of the C19–C24 ring.

**Table:**

*D*—H···*A*	*D*—H	H···*A*	*D*···*A*	*D*—H···*A*
C11—H11···O2	0.93	2.34	2.962 (2)	124
C6—H6···O3^i^	0.93	2.55	3.184 (3)	126
C22—H22···O3^ii^	0.93	2.56	3.490 (2)	174
C13—H13···*Cg*1^iii^	0.93	2.91	3.727 (2)	147

Symmetry codes: (i) *x*−1/2, −*y*+1/2, *z*+1/2; (ii) *x*−1/2, −*y*+1/2, *z*−1/2; (iii) −*x*+1, −*y*+1, −*z*.
